# The protein kinase 2 inhibitor tetrabromobenzotriazole protects against renal ischemia reperfusion injury

**DOI:** 10.1038/srep14816

**Published:** 2015-10-01

**Authors:** Sun-O Ka, Hong Pil Hwang, Jong-Hwa Jang, In Hyuk Bang, Ui-Jin Bae, Hee Chul Yu, Baik Hwan Cho, Byung-Hyun Park

**Affiliations:** 1 Department of Biochemistry, Chonbuk National University Medical School, 567 Baekje-daero, Deokjin-gu, Jeonju, Jeonbuk 54896, Republic of Korea; 2 Department of Surgery and Research Institute of Clinical Medicine, Chonbuk National University Medical School, 567 Baekje-daero, Deokjin-gu, Jeonju, Jeonbuk 54896, Republic of Korea; 3 Department of Dental Hygiene, Hanseo University, 46 Hanseo 1 ro, Seasan, Chungnam 31962, Republic of Korea

## Abstract

Protein kinase 2 (CK2) activation was reported to enhance reactive oxygen species production and activate the nuclear factor κB (NF-κB) pathway. Because oxidative stress and inflammation are critical events for tissue destruction during ischemia reperfusion (I/R), we sought to determine whether CK2 was important in the renal response to I/R. Mice underwent 25 min of renal ischemia and were then reperfused. We confirmed an increased expression of CK2α during the reperfusion period, while expression of CK2β remained consistent. We administered tetrabromobenzotriazole (TBBt), a selective CK2α inhibitor before inducing I/R injury. Mice subjected to I/R injury showed typical patterns of acute kidney injury; blood urea nitrogen and serum creatinine levels, tubular necrosis and apoptosis, inflammatory cell infiltration and proinflammatory cytokine production, and oxidative stress were markedly increased when compared to sham mice. However, pretreatment with TBBt abolished these changes and improved renal function and architecture. Similar renoprotective effects of CK2α inhibition were observed for emodin. Renoprotective effects of CK2α inhibition were associated with suppression of NF-κB and mitogen activated protein kinase (MAPK) pathways. Taken together, these results suggest that CK2α mediates proapoptotic and proinflammatory signaling, thus the CK2α inhibitor may be used to prevent renal I/R injuries observed in clinical settings.

Acute kidney injury (AKI) following ischemia reperfusion (I/R) may negatively affect the outcome of kidney transplantation. Clinically, I/R during kidney transplantation can lead to graft rejection, delayed graft function, renal cell death, and interstitial fibrosis^1^. The estimated graft survival for those with AKI is 65% vs. 85% for those without AKI^2^. Therefore, I/R injury has motivated professionals to search for alternatives that preserve the functional and morphological integrity of transplanted organs.

Ischemic insult with hypoxia and generation of reactive oxygen species (ROS) during reperfusion are believed to contribute to tissue injury^3^,^4^. ROS have direct cytotoxic effects, including DNA damage, lipid peroxidation, protein nitrosylation, and apoptosis induction^5^. ROS activate nuclear factor-κB (NF-κB), which triggers the release of a series of inflammatory mediators such as tumor necrosis factor-α (TNF-α), interleukin (IL)-1β, and IL-6^1^,^6^. At the tissue level, ROS and inflammatory cytokines activate enzymes that are involved in the processes of necrosis and apoptosis, the latter of which is most important in reperfusion injury^7^. Histologically, this manifests in disruption of the tissue lattice and interstitial edema. Antioxidants and anti-apoptotic therapy have been shown to be protective against I/R-mediated oxidative damage in different experimental models^8^,^9^,^10^,^11^.

Protein kinase 2 (CK2) is a highly conserved and ubiquitously expressed serine/threonine kinase; it is a tetramer composed of two catalytic subunits (α and α′) and two regulatory subunits (β) in an α_2_β_2_, αα′β_2_, or α′_2_β_2_ configuration^12^. CK2 is upregulated in a variety of human cancers and creates a cellular environment favorable to neoplasia by enhancing cell proliferation and by inhibiting apoptosis^13^,^14^. Thus, CK2 has emerged as a promising pharmacological target for anti-cancer therapy^15^. In addition to its apoptotic inhibiting functions, a number of studies have suggested a pro-inflammatory role for CK2. Exogenous expression of CK2α activates inhibitory κB kinase (IKK)β, which then phosphorylates and degrades inhibitory κB (IκB)α^16^. CK2α is also involved in phosphorylation of p65, which has a synergistic effect on the amplitude of transactivation^17^. Hence, CK2 inhibitors suppress NF-κB-dependent pro-inflammatory cytokine production and the related inflammatory responses^18^,^19^,^20^.

In the last two decades, a number of groups have developed various kinds of CK2 inhibitors. One of the most efficient and selective is 4,5,6,7-tetrabromobenzotriazole (TBBt). The basis for TBBt selectivity is provided by the hydrophobic pocket adjacent to the ATP/GTP binding site which is smaller in CK2, than in the majority of other protein kinases^21^. Treatment of human Jurkat cells with TBBt leads to induction of apoptosis^22^. Another CK2 inhibitor emodin, in comparison with TBBt, is more water soluble and has a limited selectivity for CK2. Besides CK2, emodin inhibits casein kinase 1^23^ and receptor tyrosine kinase^24^.

Given that apoptosis and inflammation are critical events for I/R injury, CK2 inhibition may have some role in the pathogenesis of I/R injury. Recently, Kim *et al.*^25^ demonstrated that inactivation of CK2 in the mouse brain enhances production of ROS and neuronal cell death after ischemic injury *via* increased NADPH oxidase activity. To our knowledge, however, there are no reports about its effects against renal I/R injury. Based on conflicting evidence of apoptotic induction and anti-inflammation of CK2 inhibition, we assessed the effects of TBBt on the intrinsic response to renal I/R injury.

## Results

### CK2α expression is increased during renal I/R injury

**Figure 1:**
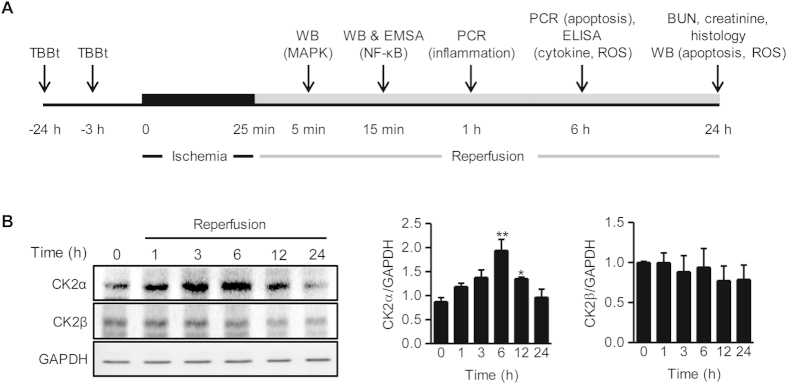
Experimental protocol and changes of CK2α expression during renal I/R injury. (**A**) Mice were intraperitoneally injected with TBBt and renal ischemia (25 min) was induced. Kidney tissues and blood samples were collected after reperfusion for each experiment. Western blotting for MAP kinases (5 min), Western blotting and EMSA for NF-κB activation (15 min), real-time RT-PCR for inflammation-related genes (1 h), ELISA (cytokines and antioxidant enzymes) and real-time RT-PCR for apoptosis (6 h), colorimetric analyses for BUN and creatinine, end-point histology (H&E, PAS, TUNEL, IHC for F4/80 and 4-HNE, and esterase staining), and Western blotting for apoptosis and oxidative stress (24 h) were performed after reperfusion. (**B**) Protein levels of CK2α and CK2β in kidney tissues were examined by Western blotting. Protein intensity was measured. Values are mean ± SEM (n = 4 mice per group).^*^p < 0.05 and ^**^p < 0.01 vs. control. WB, Western blotting; PCR, real-time RT-PCR; EMSA, electrophoretic mobility shift assay; PAS, periodic acid Schiff; 4-HNE, 4-hydroxynonenal.

To induce I/R injury, the renal pedicles were bilaterally clamped for 25 min, after which they were reperfused for various time periods (Fig. 1A). This protocol was modified from previously reported methods^4^,^26^,^27^. We first determined protein levels of CK2 in reperfused renal tissues (Fig. 1B). The protein levels of CK2α but not of CK2β began to increase 1 h after the initiation of reperfusion; they reached their maximum levels at 6 h, remained elevated up to 12 h, and declined thereafter.

### CK2α inhibition attenuates renal I/R injury

**Figure 2:**
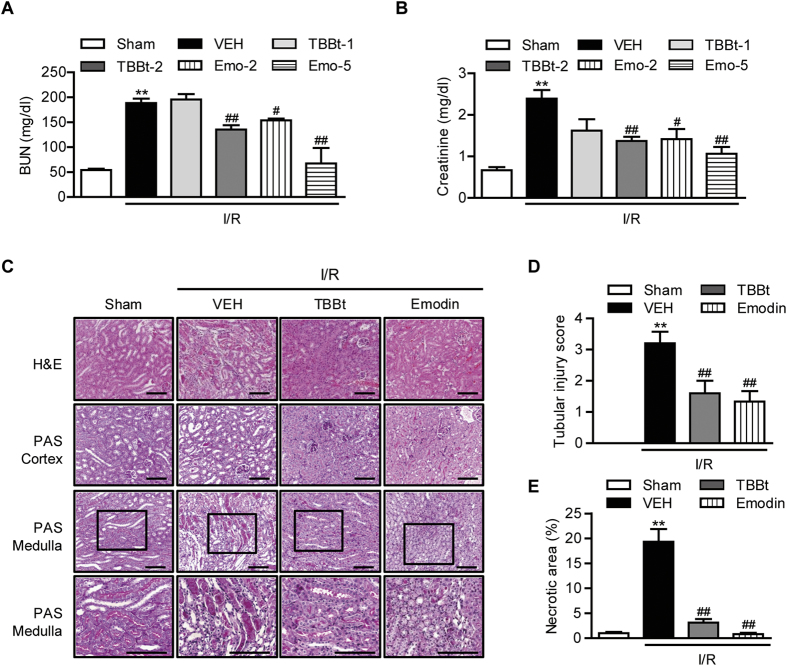
Protection of renal I/R injury by CK2α inhibitor. Mice were injected with 1 or 2 mg/kg TBBt or 2 or 5 mg/kg emodin *via* i.p. before I/R injury. After 24-h reperfusion, blood was collected for the measurement of BUN (**A**) and creatinine (**B**). (**C**) Mice were injected with 2 mg/kg TBBt or 5 mg/kg emodin, and kidney tissues were collected 24 h after reperfusion. Tissues were stained with hematoxylin and eosin (H&E, ×200) and periodic acid Schiff (PAS, ×200 or ×400) for light microscopic analysis. Bar = 250 μm. Histopathologic scoring (**D**) and quantification of necrotic area (**E**) were performed in a blind method. Values are mean ± SEM (n = 6 mice per group). ^**^p < 0.01 vs. sham; ^##^*p* < 0.01 vs. VEH. VEH, vehicle; TBBt-1, 1 mg/kg TBBt; TBBt-2, 2 mg/kg TBBt; Emo-2, emodin 2 mg/kg; Emo-5, emodin 5 mg/kg.

To assess the function of CK2α in renal I/R injury, we pretreated mice with TBBt, a CK2α inhibitor, 3 h and 24 h prior to inducing I/R injury, and blood samples were collected 24 h after reperfusion. I/R injury significantly impaired renal function in control mice as BUN and creatinine levels increased from 54.2 ± 6.2 mg/dl and 0.7 ± 0.2 mg/dl, respectively, before I/R to 198.0 ± 25.7 mg/dl and 2.4 ± 0.7 mg/dl, respectively, after reperfusion (Fig. 2A,B). However, pretreatment with 2 mg/kg TBBt significantly attenuated this impairment; the average BUN and creatinine levels in TBBt-pretreated mice were 135.4 ± 19.4 mg/dl and 1.4 ± 0.4 mg/dl, respectively (*p* < 0.01).

Macroscopically, kidneys subjected to I/R were clearly enlarged and appeared edematous (data not shown). Upon histological examination, the I/R group showed typical features of severe acute tubular damage, including extensive tubular necrosis, tubular dilatation, and loss of brush border (Fig. 2C). A scoring system was introduced to compare the severity of the histologic signs of I/R, and the tubular injury score of I/R-induced kidneys was markedly increased when compared with that of sham mice (Fig. 2D). Pretreatment with TBBt preserved the normal morphology of the kidney and resulted in slight swelling of the tubular epithelium and a slight loss of brush border. The histopathologic scores supported the histologic findings; TBBt treatment reduced the score from 3.2 ± 0.8 to 1.6 ± 0.8 (*p* < 0.01). In addition, less severe tubular necrosis was observed in TBBt-pretreated mice (Fig. 2E).

Similar renoprotective effects of CK2 inhibition were observed for emodin, another CK2 inhibitor. Emodin treatment significantly improved I/R-induced renal dysfunction (Fig. 2A,B). Emodin also significantly modulated the histological alterations observed in I/R-injured mice (Fig. 2C–E).

### TBBt decreases apoptosis after I/R injury

**Figure 3:**
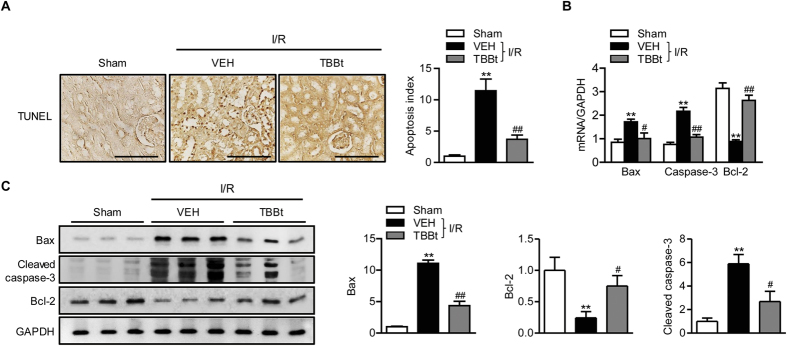
Effect of TBBt on I/R-induced apoptosis. (**A**) Mice were injected with 2 mg/kg TBBt, and kidney tissues were collected 24 h after reperfusion. Tissues were stained with TUNEL (×400). Bar = 250 μm. Apoptotic cells were counted and expressed as a percentage of all glomerular and tubular cells. The expression levels of cleaved caspase-3, Bax, and Bcl-2 were examined by real-time RT-PCR 6 h after reperfusion (**B**) or Western blotting 24 h after reperfusion (**C**). Values are mean ± SEM (n = 6 mice per group). ^*^p < 0.05 and ^**^p < 0.01 vs. sham; ^#^*p* < 0.05 and ^##^*p* < 0.01 vs. VEH.

Although the major cause of cell death during renal I/R injury is necrosis, apoptotic cell death is also observed during the reperfusion process^7^. The extent of apoptosis was evaluated by TUNEL staining (Fig. 3A). The number of TUNEL-positive apoptotic cells was markedly increased in I/R-injured mice compared to that in sham mice. Increased protein and mRNA levels of proapoptotic caspase-3 and Bax and a decreased protein level of antiapoptotic Bcl-2 were observed in I/R-injured mice (Fig. 3B,C). Pretreatment with TBBt attenuated these changes.

### TBBt decreases inflammatory cell infiltration in I/R-injured kidney tissue

**Figure 4:**
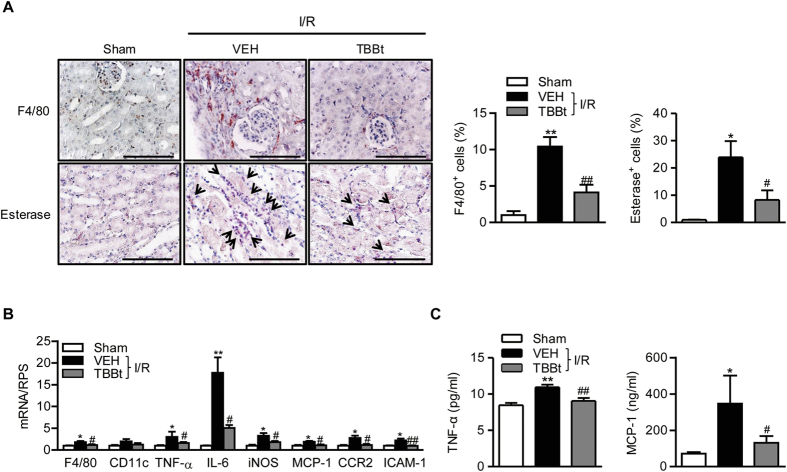
Decrease of inflammation and inflammatory mediator production by TBBt. (**A**) Mice were injected with 2 mg/kg TBBt, and kidney tissues were retrieved 24 h after reperfusion. Tissues were immunostained with antibodies against F4/80 or stained with naphthol AS-D chloroacetate esterase. Neutrophils are colored red. Bar = 250 μm. (**B**) Expression of inflammation-related genes was determined by real-time RT-PCR 1 h after reperfusion. (**C**) Plasma levels of TNF-α and MCP-1 were determined by ELISA 6 h after reperfusion. Values are mean ± SEM (n = 6 mice per group). ^*^p < 0.05 and ^**^p < 0.01 vs. sham; ^#^*p* < 0.05 and ^##^*p* < 0.01 vs. VEH.

The inflammatory response following I/R injury is related to renal dysfunction^1^. To investigate whether improved renal function in TBBt-pretreated mice was dependent on a suppressed inflammatory reaction, we stained kidney tissues with F4/80 and naphthol AS-D chloroacetate esterase, which are specific for macrophages and neutrophils, respectively. After 24 h of reperfusion, the number of infiltrating macrophages and neutrophils were increased in the I/R-injured kidney tissues, whereas significantly less infiltration of macrophages and neutrophils was observed in TBBt-pretreated mice (Fig. 4A). Real-time RT-PCR analysis confirmed the decreased accumulation of macrophages and suppressed inflammation in TBBt-pretreated mice compared to I/R mice (Fig. 4B). Changes in serum TNF-α and MCP-1 levels were similar to those seen for mRNA expression (Fig. 4C).

### TBBt inhibits NF-κB signaling pathway

**Figure 5:**
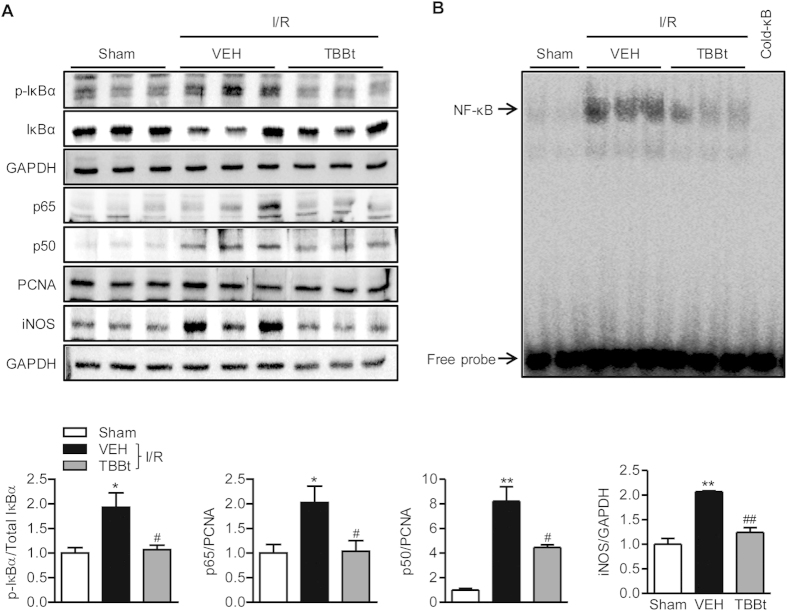
Suppression of NF-κB signaling pathway by TBBt. (**A**) Mice were injected with 2 mg/kg TBBt, and total lysate and nuclear extract were prepared from kidney homogenates. Nuclear translocation of p65 and p50 subunits and phosphorylation of cytoplasmic IκBα were analyzed by Western blotting 15 min after reperfusion. The expression level of iNOS in total lysate was analyzed by Western blotting 24 h after reperfusion. (**B**) Nuclear proteins were prepared and DNA binding of p65 subunit was analyzed using EMSA15 min after reperfusion. ^*^p < 0.05 and ^**^p < 0.01 vs. sham; ^#^*p* < 0.05 and ^##^*p* < 0.01 vs. VEH.

We next investigated the effects of TBBt on I/R-dependent NF-κB activation, which is a major signaling pathway of inflammation. In I/R mice, there were increases in nuclear p65 and p50 subunits and cytoplasmic p-IκBα compared to sham mice (Fig. 5A). Accordingly, expression of iNOS, a downstream effector of NF-κB, was also increased in I/R mice. In contrast, in the presence of TBBt, these changes were almost completely inhibited. Further examination by EMSA clearly showed nuclear translocation of p65 and binding to an NF-κB consensus sequence in I/R mice but not in TBBt-pretreated mice (Fig. 5B). Our data suggest that TBBt could successfully inhibit NF-κB activation induced by renal I/R.

### TBBt inhibits the MAPK signaling pathway

**Figure 6:**
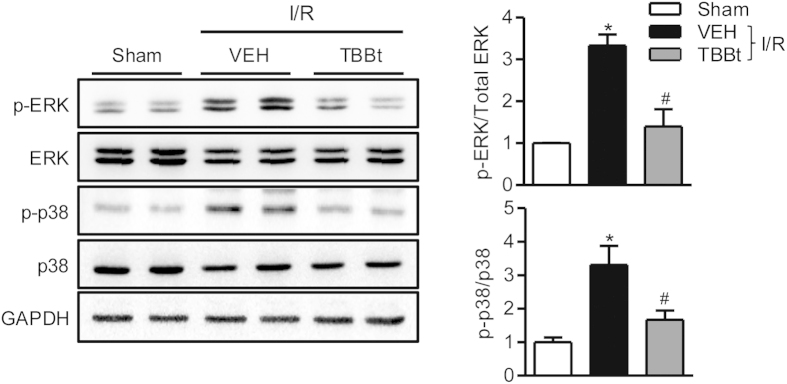
Inhibition of ERK and p38 MAPK signaling pathways by TBBt. Mice were injected with 2 mg/kg TBBt, and total lysates were prepared from kidney tissues 5 min after reperfusion. Expression and phosphorylation levels of p38 MAPK and ERK were analyzed by Western blotting. Values are expressed as the mean ± SEM (n = 5 mice per group). ^*^p < 0.05 vs. sham; ^#^*p* < 0.01 vs. VEH.

To investigate the mechanism by which TBBt suppresses the NF-κB signaling pathway, we compared mitogen-activated protein kinase (MAPK) signal transduction pathways. I/R injury increased the phosphorylation levels of ERK and p38 MAPK in I/R-injured kidney tissues but not in those of TBBt mice (Fig. 6). Phosphorylation of JNK was not different between groups (data not shown).

### TBBt prevents the down-regulation of anti-oxidant potentials

**Figure 7:**
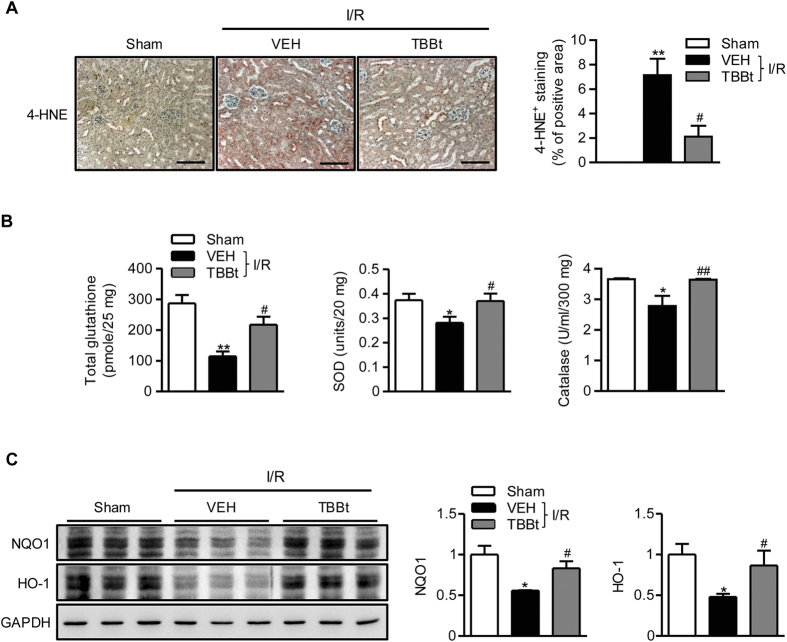
Attenuation of I/R-induced oxidative stress by TBBt. (**A**) Mice were injected with 2 mg/kg TBBt, and kidney tissues were stained with 4-HNE 24 h after reperfusion. The area of 4-HNE positive cells was analyzed. Values are expressed as the mean ± SEM (n = 4–6 mice per group). ^**^p < 0.01 vs. sham; ^#^*p* < 0.05 vs. VEH. (**B**) Tissue levels of GSH, SOD, and catalase were analyzed using ELISA 6 h after reperfusion. Values are expressed as the mean ± SEM (n = 12 mice per group). ^*^p < 0.05 and ^**^p < 0.01 vs. sham; ^#^*p* < 0.05 and ^##^*p* < 0.01 vs. VEH. (**C**) Kidney tissues were retrieved 24 h after reperfusion, and NQO1 and HO-1 levels were examined by Western blotting. Values are expressed as the mean ± SEM (n = 6 mice per group). ^*^p < 0.05 vs. sham; ^#^*p* < 0.05 vs. VEH.

I/R injury is partially mediated by oxidative stress^3^, and CK2 inhibition is known to increase oxidative stress under *in vitro* cell culture condition^28^. We investigated whether TBBt increases oxidative stress. Surprisingly, TBBt pretreatment caused suppression of ROS production as observed by decrease in 4-hydroxynonenal (4-HNE) positive cells (Fig. 7A). Compared with I/R mice, increases in glutathione levels and superoxide dismutase and catalase enzyme activities were observed in TBBt-pretreated mice (Fig. 7B), suggesting increased antioxidant potentials. To explain increased anti-oxidant potentials by TBBt, we compared the downstream signaling pathway of NF-E2-related factor-2 (Nrf2), a master regulator antioxidant response, in treated and control mice. Compared to control mice, TBBt pretreatment led to increases in expression levels of NAD(P)H quinone oxidoreductase (NQO1) and hemeoxygenase (HO)-1 (Fig. 7C). These results indicate that TBBt protects kidney I/R injury through preventing the down-regulation of antioxidant genes after I/R injury.

## Discussion

In this study, we demonstrated for the first time that pretreatment with a double dose of CK2α inhibitor TBBt significantly protected against renal I/R injury. After bilateral renal I/R injury, TBBt pretreated mice displayed significantly preserved renal function, less histological tubular damage, and reduced inflammatory cell infiltration. The protective effect was associated with ameliorated oxidative stress as manifested by an increase in antioxidant capacity (a higher level of glutathione, higher enzyme activities of SOD and catalase, and lower production of 4-HNE). We further demonstrated that TBBt reduced NF-κB activation and concomitant inflammatory cytokine production through inhibition of the ERK and p38 MAPK pathways.

I/R-injured kidney tissue is characterized by inflammatory cell infiltration, initially with neutrophils and later with macrophages and lymphocytes^1^. Ischemic injury upregulates the expression of various inflammation-related molecules such as MCP-1 and ICAM-1, which in turn facilitate the recruitment and infiltration of inflammatory cells in the kidney tissue^9^,^29^,^30^. Macrophages constitute 40–60% of infiltrating cells during acute allograft rejection. In patients with acute rejection, interstitial macrophage infiltration was significantly higher than in non-rejecting patients^31^. Recently, Kim *et al.*^18^ reported that CK2α is involved in the platelet activating factor-induced NF-κB activation and that inhibitors for each step block inflammatory cell infiltration into tissues. An increase of CK2α expression and inflammatory cell infiltration in a murine glomerulonephritis model has also been reported to lead to increases in production from genes in the ERK pathway (c-fos, Egr-1, and Elk-1), NF-κB-regulated inflammatory cytokines (TNF-α and MCP-1), and extracellular matrix proteins (collagen, fibronectin, TGFβ, and PDGF)^32^. Inhibition of CK2α prevented inflammation and decreased expression of the aforementioned genes. These reports point to an important role of CK2α expression for inflammatory cell infiltration during I/R injury. In line with these reports, we observed increased expression of CK2α, MCP-1, and ICAM-1 and activation of the NF-κB pathway in I/R injured kidney tissues. Because MCP-1 is a potent macrophage chemoattractant, increased levels of MCP-1 with its receptor CCR2 could directly trigger the recruitment of macrophages to kidney tissue. Infiltrated macrophages could, in turn, secrete a variety of cytokines, including TNF-α and IL-6, which would further induce inflammation-related gene expression and promote local inflammatory responses^33^. Expression of the NF-κB downstream protein iNOS has also been linked to graft rejection in a rat model of allogenic kidney transplantation, where macrophage depletion resulted in a reduction of acute rejection and an upregulation of iNOS expression^34^. In this study, we provided evidence that all of these inflammatory events are associated with CK2α activation, because TBBt effectively suppressed NF-κB activation, cytokine production, and inflammatory cell infiltration in I/R injured kidney tissues. Ultimately, CK2α suppression seems to contribute to decrease of apoptotic or necrotic cell death.

CK2 is strongly implicated in regulation of cell proliferation and in inhibition of apoptosis^13^,^14^. However, results vary greatly depending on the cell type and stress model. For example, activation of the proapoptotic pathway by CK2α has been observed in INS-1 cells and isolated islets^35^. Likewise, in our study, CK2α inhibition led to anantiapoptotic effects in renal tubular tissues, which exhibited less tubular injury and enhanced clearance of BUN and creatinine. Since TNF-α has been shown to be an important mediator of tubular epithelial cell apoptosis^36^,^37^, we suspected that TBBt might have antiapoptotic effects through suppression of the TNF-α-mediated apoptosis pathway. Indeed, we observed significantly suppressed levels of TNF-α mRNA and protein in TBBt-pretreated mice.

Our results are in contrast to those of Kim *et al.*^25^ who demonstrated that CK2α was a negative regulator of NADPH oxidase, an important enzyme for ROS generation in the mouse brain. They further found that the protein levels and enzyme activities of CK2α were significantly reduced after transient focal cerebral I/R injury, and CK2α inhibitor suppressed the death of primary neuronal cells exposed to hypoxia and reoxygenation injury. These opposite conclusions may result from several differences in the two studies. First, each study used a different CK2α inhibitor (TBBt for our study vs. TBCA for theirs). Schneider *et al.*^38^ compared two CK2α inhibitors, 4,5,6,7-tetrabromobenzimidazole (TBBz) and 2-dimethylamino-4,5,6,7-tetrabromo-1H-benzimidazole (DMAT) and found that both chemicals inhibited CK2 activity to the same extent, but only DMAT induced ROS generation. Their report suggests that CK2 inhibition is not a prerequisite for ROS generation and that the two closely related CK2α inhibitors may activate different signaling pathways for ROS generation. Second, we used a kidney model with ischemia injury for 25 min using ketamine as an anesthesia, whereas Kim *et al.* experimented on a brain model with 45 min of ischemia using isoflurane as the anesthesia. Third, even using different strains of mice (C57BL/6 in our study vs. CD1 in theirs) could have contributed to the different outcomes. Fourth, in contrast to the brain I/R model, we did not observe a suppression of CK2α expression after reperfusion; rather, we observed an increase in CK2α (Fig. 1B). Taken together, differences in experimental design could lead to the ultimate beneficial or deleterious outcome for CK2α inhibition in I/R injury.

Collectively, our results demonstrate that CK2α inhibition protects against renal I/R injury by the following mechanism: inhibition of p38 and ERK pathways → suppression of NF-κB pathway → attenuation of inflammation and oxidative stress → prevention of apoptosis and concomitant renal dysfunction. Thus, CK2α may have an aggravating role in the pathogenesis of renal I/R injury.

## Materials and Methods

### Reagents

TBBt was purchased from Tocris (Bristol, UK) and dissolved at 50 mg/ml in dimethyl sulfoxide. All reagents were purchased from Sigma-Aldrich (St. Louis, MO, USA) unless otherwise noted. Emodin was isolated from *R. palmatum* as described previously^39^.

### Animal experiment

Pathogen-free 7–9-week-old C57BL/6 male mice (Damul Science, Daejeon, Republic of Korea; weight 20–23 g) were maintained on a diet of standard laboratory chow and water *ad libitum*. Mice were treated with TBBt (1 or 2 mg/kg body weight in 200 μl of 5% DMSO in saline), emodin (2 or 5 mg/kg body weight 200 μl of 5% DMSO in saline), or the same volume of vehicle through i.p. injection at 3 h and 24 h before ischemia. Mice were anaesthetized with a ketamine-xylazine mixture through i.m. injection. In the I/R group, both renal pedicles were clamped through midline incisions using a microvascular clip^40^. The kidney was kept moist with gauze soaked in saline, and body temperature was maintained at 37 °C with a warm blanket throughout ischemia. After 25 min of ischemia, the clip was removed to initiate reperfusion. Sham mice underwent the same operation without vascular occlusion. After the desired reperfusion time, mice were killed, and serum and tissue samples were collected and immediately fixed in 10% formalin or stored at −80 °C until further analysis. All animal experiments were performed in accordance with the Guide for the Care and Use of Laboratory Animals, published by the US National Institutes of Health (NIH Publication No. 85–23, revised 2011). The current study protocol was also approved by the Institutional Animal Care and Use Committee of Chonbuk National University (Approval No. CBNU 2015–051).

### Biochemical analysis

Blood samples were collected 24 h after reperfusion. Blood urea nitrogen (BUN) and serum creatinine were measured using specific assay kits (Arbor Assays, Ann Arbor, MI, USA). TNF-α (Invitrogen, Carlsbad, CA, USA) and monocyte chemotactic protein 1 (MCP-1, R&D Systems, Minneapolis, MN, USA) were measured using specific ELISA kits.

### Anti-oxidant enzyme activity assay

To analyze the enzymatic activities of superoxide dismutase (SOD), catalase, and glutathione (GSH), kidney tissues were suspended in 10 mM phosphate buffer (pH 7.4), mixed with ice-cold 5% metaphosphoric acid solution and then homogenized. Homogenates were centrifuged at 3000 rpm for 10 min. Enzyme activity in the supernatant was determined using commercial assay kits (Enzo Life Sciences, Plymouth Meeting, PA, USA).

### Histology and histopathologic scoring

Kidney tissues were fixed in 10% neutral buffered formalin and then embedded in paraffin. Tissue was sectioned at 4 μm and then stained with hematoxylin and eosin (H&E) and periodic acid-Schiff reagent (PAS) for light microscopic analyses. Histopathologic damage was defined as tubular epithelial swelling, loss of brush border, vacuolar degeneration, necrotic tubules, cast formation, and desquamation. The degree of tubular injury was estimated at a ×200 magnification using 5 randomly selected fields for each kidney with the following standard; 0, normal; 1, damage involving <25% of tubules; 2, damage involving 25–50% of tubules; 3, damage involving 50–75% of tubules; and 4, damage involving 75–100% of tubules^40^. Tubular necrosis was quantitated as the percentage of tubules in the outer medulla in which epithelial necrosis or necrotic debris was observed in PAS-stained sections.

### Neutrophil infiltration

A naphthol AS-D chloroacetate esterase kit (Sigma-Aldrich) was used for neutrophil esterase staining of kidney sections. Neutrophils were counted by examining 5–10 viewing fields randomly selected from the outer medulla and corticomedullary junction on each slide at ×400 magnification in a blinded manner. The number of neutrophils were counted by iSolution DT 36 software (Carl Zeiss, Oberkochen, Germany), and results were expressed as percentage of the total cells.

### Immunohistochemistry

Immunohistochemical staining was performed using the DAKO Envision system (DAKO, Carpinteria, CA, USA), which uses dextran polymers conjugated with horseradish peroxidase to avoid contamination with endogenous biotin. After deparaffinization, tissue sections were treated using a microwave antigen-retrieval procedure in 10 mM sodium citrate buffer. After blocking endogenous peroxidases, sections were incubated with Protein Block Serum-Free (DAKO) to block non-specific staining and were immunostained with antibody against F4/80 or 4-hydroxynonenal (both from Abcam, Cambridge, UK). Stained sections were quantified by iSolution DT 36 software (Carl Zeiss, Oberkochen, Germany), and results were expressed as percentage of the total cells.

### TUNEL assay

TUNEL staining was used to detect apoptotic cells (Promega, Madison, WI, USA). Apoptotic cells were counted under a microscope (×200) and expressed as the apoptosis index (AI = number of apoptotic bodies/100 cells). Each group was assessed in triplicate, and data were averaged.

### Protein extraction

Total protein of kidney tissue was extracted using a tissue protein extraction reagent (Pierce Biotechnology, Rockford, IL, USA). For nuclear protein extraction, kidney tissues were homogenized with a pre-chilled Dounce homogenizer (Wheaton Industries, Millville, NJ, USA) and then extracted with nuclear and cytoplasmic extraction reagents (Pierce Biotechnology). Protein concentration was determined using the Bradford method.

### Electrophoretic mobility shift assay (EMSA)

Nuclear extracts prepared from the kidney tissues were incubated with proteinase inhibitor cocktail (Calbiochem, Billerica, MA, USA). An oligonucleotide corresponding NF-κB site (5-CCGGTTAACAGAGGGGGCTTTCCGAG-3) was synthesized and used as a probe for a gel retardation assay. The two complementary strands were annealed and labeled with [α-^32^P]dCTP. Labeled probe (10,000 cpm), 10 μg of nuclear extract, and binding buffer (10 mMTris-HCl, pH 7.6, 500 mM KCl, 10 mM EDTA, 50% glycerol, 100 ng poly(dI·dC), and 1 mM dithiothreitol) were then incubated for 30 min at room temperature in a final volume of 20 μl. Reaction mixtures were analyzed by electrophoresis on 4% polyacrylamide gels in 0.5× Tris-borate buffer. Gels were dried and examined by autoradiography.

### Western blotting

Homogenates containing 20 μg of total protein were separated by SDS-PAGE and transferred to nitrocellulose membranes. Blots were probed with primary antibody against CK2α, CK2β, HO-1, NQO1, IκBα, iNOS, PCNA, p65 and p50 (Santa Cruz Biochemicals, Dallas, TX, USA) or Bax, Bcl2, p-IκBα, p-ERK, ERK, p-p38 and p38 (Cell Signaling, Danvers, MA, USA) and signals were detected with a Las-4000 imager (GE Healthcare Life Science, Pittsburgh, PA, USA).

### RNA isolation and real-time RT-PCR

Total RNA was extracted from frozen kidney tissue using the RNA Iso kit (Takara, Shiga, Japan). RNA was precipitated with isopropanol and dissolved in diethylpyrocarbonate-treated distilled water. Total RNA (1 μg) was treated with RNase-free DNase (Invitrogen), and first-strand cDNA was generated using the random hexamer primer provided in the first-strand cDNA synthesis kit (Invitrogen). Specific primers for each gene were designed using qPrimerDepot (http://mouseprimerdepot.nci.nih.gov, Supplementary Table 1). Glyceraldehyde 3-phosphate dehydrogenase (GAPDH) was used as an invariant control. The real-time RT-PCR reaction mixture consisted of 10 ng reverse transcribed RNA, 200 nM forward and reverse primers, and 2× PCR master mixture in a final volume of 10 μl. The PCR reaction was carried out in 384-well plates using the ABI Prism 7900HT Sequence Detection System (Applied Biosystems, Foster City, CA, USA).

### Statistical analysis

Statistical analyses of the data were conducted using ANOVA and Duncan’s tests. Differences with a *p* < 0.05 were considered statistically significant.

## Additional Information

**How to cite this article**: Ka, S.-O. *et al.* The protein kinase 2 inhibitor tetrabromobenzotriazole protects against renal ischemia reperfusion injury. *Sci. Rep.* **5**, 14816; doi: 10.1038/srep14816 (2015).

## Supplementary Material

Supplementary Information
