# Supplementary material for: The protein kinase 2 inhibitor tetrabromobenzotriazole protects against renal ischemia reperfusion injury
Source: Sci Rep. 2015 Oct 1;5:14816. doi: 10.1038/srep14816 (PMC4589787; doi:10.1038/srep14816)
Supplement: Supplementary Information [file srep14816-s1.pdf]

The protein kinase 2 inhibitor tetrabromobenzotriazole protects against renal ischemia reperfusion injury

Sun-O Ka[a,*], Hong Pil Hwang[b,*], Jonghwa Jang[c,*], In Hyuk Bang[a], Ui-Jin Bae[a], Hee Chul Yu[b], Baik Hwan Cho[b], and Byung-Hyun Park[a,¶]

Departments of [a]Biochemistry and [b]Surgery, Chonbuk National University Medical School, 567 Baekje-daero, Deokjin-gu, Jeonju, Jeonbuk 54896, Republic of Korea

[c]Department of Dental Hygiene, Hanseo University, 46 Hanseo 1 ro, Seasan, Chungnam 31962, Republic of Korea

Table S1. Sequences and accession numbers for primers (FOR, forward and REV, reverse) used in real time RT-PCR.

Gene	Sequences for primers	Accession NO.
Bax	FOR: GATCAGCTCGGGCACTTTAG<br>REV: TTGCTGATGGCAACTTCAAC	NM_007527
Caspase-3	FOR: GACTTGCTCCCATGTATGGTC<br>REV: ATCAAAGCGCAGTGTCCTG	NM_009810
Bcl-2	FOR: GGTCTTCAGAGACAGCCAGG<br>REV: GATCCAGGATAACGGAGGCT	NM_009741
iNOS	FOR: TTCTGTGCTGTCCCAGTGAG<br>REV: TGAAGAAAACCCCTTGTGCT	NM_010927
TNF-α	FOR: AGGGTCTGGGCCATAGAACT<br>REV: CCACCACGCTCTTCTGTCTAC	NM_013693
IL-6	FOR: ACCAGAGGAAATTTTCAATAGGC<br>REV: TGATGCACTTGCAGAAAACA	NM_031168
F4/80	FOR: TTTCCTCGCCTGCTTCTTC<br>REV: CCCCGTCTCTGTATTCAACC	NM_010130
CD11c	FOR: CACTCAGTGACTGCCCAAAA<br>REV: CCTCAAGACAGGACATCGCT	NM_021334
MCP-1	FOR: ATTGGGATCATCTTGCTGGT<br>REV: CCTGCTGTTCACAGTTGCC	NM_011333
CCR2	FOR: AGCACATGTGGTGAATCCAA<br>REV: TGCCATCATAAAGGAGCCA	NM_009915
ICAM-1	FOR: AACAGTTCACCTGCACGGAC<br>REV: GTCACCGTTGTGATCCCTG	NM_010493
GAPDH	FOR: CGTCCCGTAGACAAAATGGT<br>REV: TTGATGGCAACAATCTCCAC	NM_008084
